# Olfactory responses of *Trissolcus mitsukurii* to plants attacked by target and non-target stink bugs suggest low risk for biological control

**DOI:** 10.1038/s41598-022-05873-w

**Published:** 2022-02-03

**Authors:** Gabriele Rondoni, Elena Chierici, Lucrezia Giovannini, Giuseppino Sabbatini-Peverieri, Pio Federico Roversi, Eric Conti

**Affiliations:** 1 Department of Agricultural, Food and Environmental Sciences, University of Perugia, Perugia, Italy; 2 CREA – Research Centre for Plant Protection and Certification, Florence, Italy

**Keywords:** Behavioural ecology, Evolutionary ecology, Invasive species, Animal behaviour, Entomology

## Abstract

In crop systems, successful management of invasive insect herbivores can be achieved through the introduction of exotic biocontrol agents, parasitoids or predators, having a coevolutionary history with the pest. To avert threats to local biodiversity, recent legislations require a risk assessment for the organism to be released. Evaluation of its ability to exploit, for host location, odours associated with target and non-target species is crucial for a better definition of its ecological host range. Using Y-tube olfactometer bioassays in a quarantine laboratory, we investigated the ability of the Asian egg parasitoid *Trissolcus mitsukurii* (Hymenoptera: Scelionidae) to exploit odours associated with the global invader *Halyomorpha halys* (Hemiptera: Pentatomidae) and with non-target stink bugs native to Southern Europe. We demonstrated that *T. mitsukurii* is attracted by plants exposed to feeding and egg deposition of the coevolved *H. haly*s and the native *Nezara viridula*, while it is not attracted by physogastric (gravid) females or eggs alone. Remarkably, *T. mitsukurii* is repelled by plants bearing eggs of the beneficial *Arma custos*. Our results contribute to a more thorough and nuanced assessment of the potential non-target risks in the case of mass-release of parasitoids as part of a biological control programme for invasive stink bugs.

## Introduction

Management of invasive arthropod species in agricultural settings encompasses multiple strategies, including the introduction and release of exotic natural enemies^1^. The release of a biocontrol agent that is coevolved with the target herbivore allows the restoration of ecological processes, with positive implications for pest suppression^2^. On the other hand, biocontrol agents can have negative effects on the community of native natural enemies and non-target herbivores, with consequences for local biodiversity that can be hard to foresee^3^. Therefore, in the process of selecting a candidate biological control agent, a careful selection process with detailed bioassays is crucial for maximizing the benefits of pest control and, at the same time, minimizing ecological risks^4^. Recent national and international regulations require that applications for licensing candidate biocontrol agents are supported by thorough risk assessment documentation^5^. Simple evaluation of the physiological host range provides a first understanding of whether a natural enemy will be suitable for use as a biocontrol agent of a given target pest^6,7^. Such protocols typically encompass no-choice and choice laboratory bioassays in small settings, i.e., Petri dish arenas, or larger insect cage tests^8^. However, effective interactions of the introduced natural enemy in the field and the general impact on local insect communities depend on many ecological factors. Among these, the ability of the natural enemy to exploit odours from plants attacked by insects (synomones) and/or host instars (kairomones) at a long-range distance^9^. In the hierarchical process of host/prey location, these odours can help the natural enemy to efficiently locate the host^10,11^. But, when dealing with exotic species, a lack of coevolutionary history between members of the plant-host-parasitoid tritrophic system is expected, and may imply that host-associated odours are not reliable cues for parasitoids^12,13^. Therefore, incorporating evaluation of odour attractiveness in pre-release risk assessment can be crucial for determining the likelihood of species interactions in open field conditions^14,15^. Y-tube olfactometer bioassays have been demonstrated to be very well suited for evaluating parasitoid response to odours^15–19^. Here, we explored this concept on an exotic egg parasitoid as a candidate biocontrol agent of an invasive stink bug and conducted olfactometer bioassays to investigate behavioural responses towards odours associated with target and non-target species.

The brown marmorated stink bug, *Halyomorpha halys* Stål (Hemiptera: Pentatomidae), is an economically important invasive pest of major concern worldwide^20^. Its aptitude to aggregate and hide in small spaces, e.g., shipping containers, facilitates its worldwide spread^21^. Native to East Asia, this species was accidentally introduced in the US and first detected in 1996^22^. The feeding on many plant species, including a large number of crops and fruit plants, caused about US $37 million losses to the apple crop in the US in 2010^20^. In Europe, *H. halys* was first observed in 2004 (Switzerland) and since then discoveries have multiplied in European countries, including Italy^23,24^. After its establishment in Italy, *H. halys* has damaged field crops and orchards, causing serious economic damage with fruit losses that were estimated at nearly EUR 600 millions in 2019^25,26^.

Current management strategies against *H. halys* include chemical, biotechnological and physical control, while classical and augmentative biological control are promising methods under investigation^27,28^. The most effective natural enemy of *H. halys* appears to be the Asian egg parasitoid *Trissolcus japonicus* (Ashmead) (Hymenoptera: Scelionidae), which exhibits high field parasitism rates in its area of origin^29,30^. Adventive populations of *T. japonicus* have been documented in North America and Europe, where this species co-occurs with *H. halys* in some of the areas invaded by the stink bug^8,28,31–33^. Additionally, adventive populations of another Asian egg parasitoid species, *Trissolcus mitsukurii* (Ashmead) (Hymenoptera: Scelionidae), have been recently detected in Northern Italy on *H. halys* egg masses^33–35^. Frequently described as a native parasitoid of *Nezara viridula* L. (Hemiptera: Pentatomidae) in Japan^36,37^, *T. mitsukurii* also parasitizes *H. halys* and other stink bug species in China and Japan^38–40^. In Italy, adventive populations of *T. mitsukurii* have shown high parasitism efficacy on *H. halys*, comparable to that of *T. japonicus*^35^. Considering this, *T. mitsukurii* represents a promising egg parasitoid of *H. halys* in Italy and neighbouring territories^26,41^, and is currently under evaluation for the development of a pre-emptive biological control program of the stink bug in Australia and New Zealand^42^.

A detailed screening of *T. mitsukurii* host range using choice and no-choice experiments in Petri dishes was recently conducted and revealed oligophagy of this parasitoid limited to members of the Pentatomoidea superfamily^43^. However, the capability of this parasitoid to exploit odours from the plant-host system for locating stink bug eggs in the field is unknown. Results from previous studies revealed that *Trissolcus* spp. are primarily attracted by oviposition-induced plant synomones^44–48^. Here we hypothesized that the oligophagous behaviour shown by *T. mitsukurii* during assessment of its physiological host range^43^ will be shown to be narrower with the use of chemical ecology bioassays. We hypothesize that only odours associated with those host species that exhibit coevolutionary history with *T. mitsukurii* would elicit behavioural responses of the parasitoid.

Therefore, we conducted olfactometer bioassays and tested whether the parasitoid responds differently to odours from plants exposed to oviposition of *H. halys* or non-target native stink bugs, specifically *Arma custos* F., *Dolycoris baccarum* L., *Eurydema ventralis* Kolenati (Hemiptera: Pentatomidae) and *N. viridula*. Odours from physogastric (gravid) females and egg masses alone were also tested. Of the different species, *H. halys* and *A. custos* naturally occur in *T. mitsukurii*’s native area, hence it is likely that a certain level of coevolution has occurred^49,50^. The other stink bugs evaluated, *D. baccarum*, *E. ventralis* and *N. viridula*, are of West Palearctic or Mediterranean origins. They occur in part of Asia, where their presence should be considered outside their native range^51,52^.

Results of this investigation would help to understand the possible ecological impact of the exotic parasitoid *T. mitsukurii* in novel ecosystems. Additionally, they will provide foundational data for the preparation of a risk-assessment document, in support of a petition for releasing the parasitoid in areas that are seriously infested by *H. halys*.

## Results

### Behavioural responses to odours from plants bearing an egg mass

**Figure 1:**
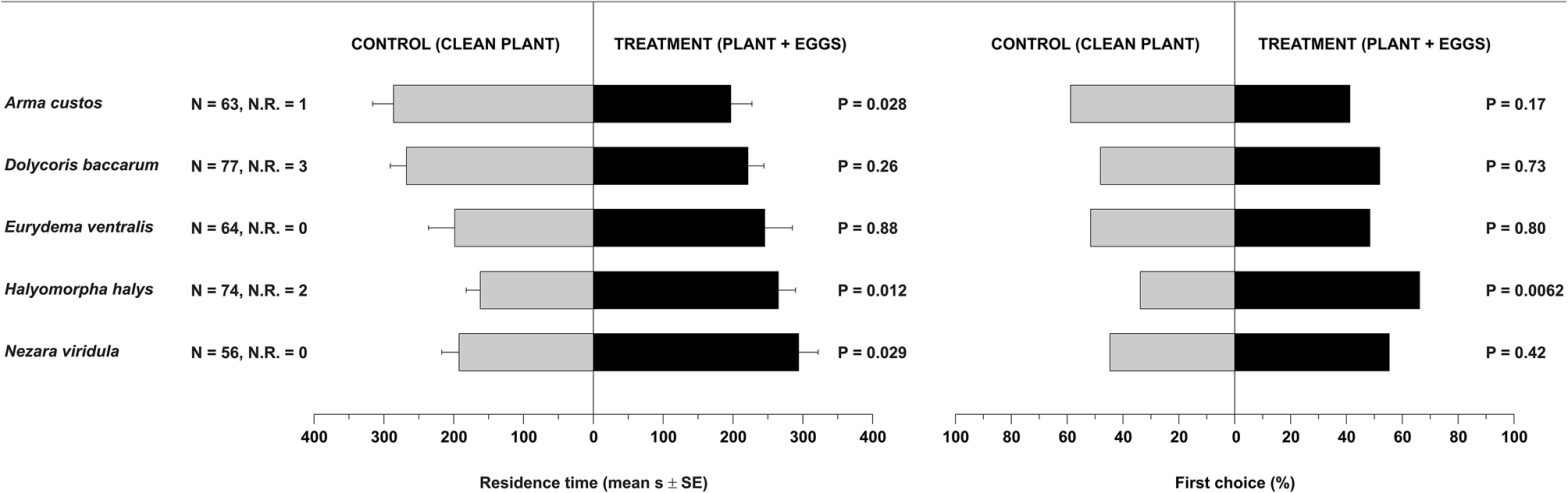
Residence time (means ± SE) and first choice (%) of *Trissolcus mitsukurii* females in Y-tube olfactometer exposed to odours from stink bug–plant systems. Treatments consisted of volatiles from soybean plants bearing an egg mass of *Arma custos*, *Dolycoris baccarum*, *Halyomorpha halys*, or *Nezara viridula*, or volatiles from cauliflower plants bearing an egg mass of *Eurydema ventralis*. Control consisted of a clean soybean or cauliflower plant. *N* number of responding insects. *N.R.* number of not-responding insects (discarded from the analysis). Planned comparisons were tested within GLM with Gaussian error distribution (residence time) or with binomial error distribution (first choice).

*Trissolcus mitsukurii* females responded positively to odours associated with the target host (soybean plant with an egg mass of *H. halys*) as their residence time in the treatment arm was higher compared to that in the control arm (contrast result for Gaussian GLM: *P* = 0.012) (Fig. 1, Table S1). Similarly, *T. mitsukurii* was attracted (higher residence time compared to control) to odours associated with *N. viridula* (soybean plant with an egg mass) (*P* = 0.029). Notably, parasitoids appeared to avoid odours associated to *A. custos* (soybean with an egg mass), displaying a lower residence time in treatment vs. control arm (*P* = 0.028). In contrast, *T. mitsukurii* did not respond to odours associated with *D. baccarum* (soybean plant with an egg mass) or *E. ventralis* (cabbage plant with an egg mass), as residence time was similar between control and treatments (*P* ≥ 0.26 for both comparisons). First choice data confirmed the preference of female egg parasitoids for plants carrying *H. halys* eggs (contrast results for binomial GLM: *P* = 0.0062) (Fig. 1, Table S2). Conversely, first choices were similar between control and treatments for all the other tested species (*P* ≥ 0.17).

### Behavioural responses to odours from stink bug females

**Figure 2:**
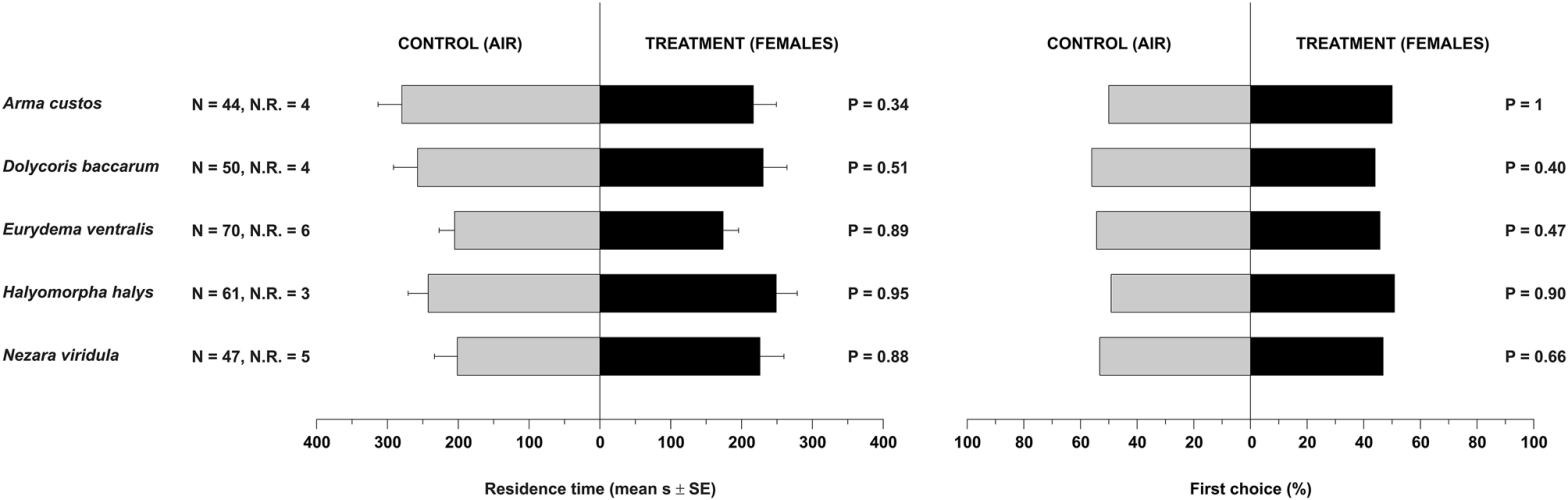
Residence time (means ± SE) and first choice (%) of *Trissolcus mitsukurii* females in Y-tube olfactometer exposed to odours from stink bug females. Treatments consisted of volatiles from females of *Arma custos*, *Dolycoris baccarum*, *Eurydema ventralis*, *Halyomorpha halys*, or *Nezara viridula*. Control consisted of clean air. *N* number of responding insects. *N.R.* number of not-responding insects (discarded from the analysis). Planned comparisons were tested within GLM with Gaussian error distribution (residence time) or with binomial error distribution (first choice).

*Trissolcus mitsukurii* females did not prefer *H. halys* (*P* = 0.95) nor those of the native species *A. custos*, *D. baccarum*, *E. ventralis* and *N. viridula* females as residence time was similar in the treatment and control arms (*P* ≥ 0.34 for all the comparisons) (Fig. 2, Table S3). Correspondingly, first choice data did not differ between control and treatments (*P* ≥ 0.40 for all comparisons) (Fig. 2, Table S4).

### Behavioural responses to odours from stink bug eggs

**Figure 3:**
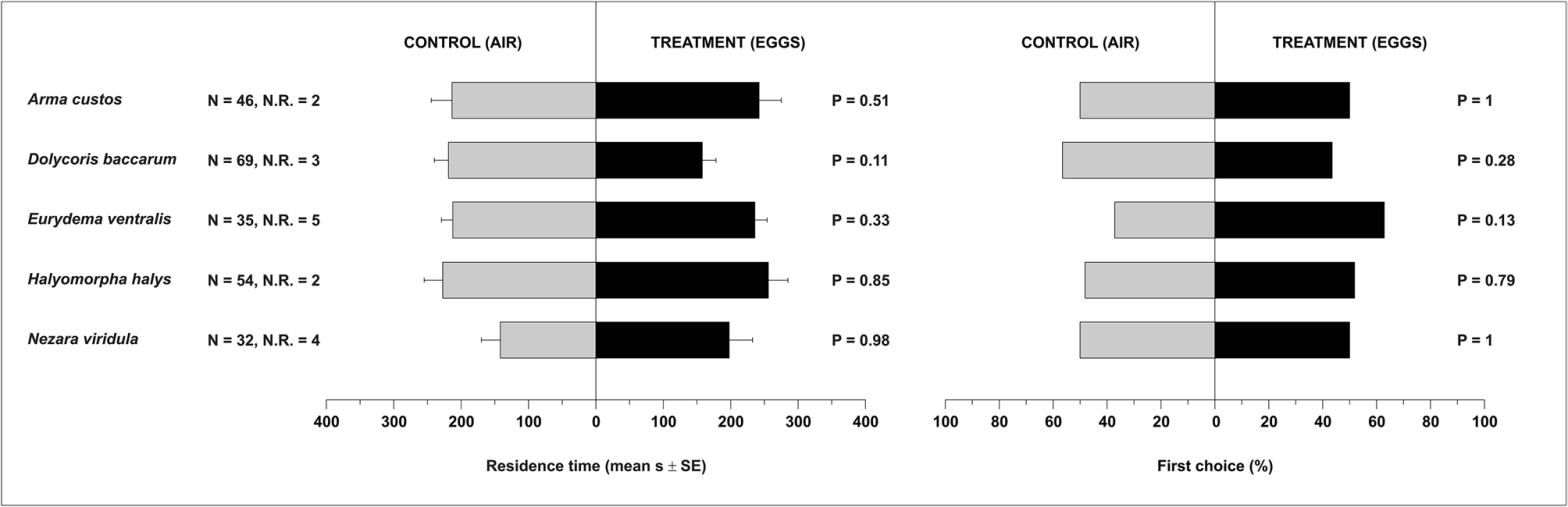
Residence time (means ± SE) and first choice (%) of *Trissolcus mitsukurii* females in Y-tube olfactometer exposed to odours from stink bug eggs. Treatments consisted of volatiles from eggs of *Arma custos*, *Dolycoris baccarum*, *Eurydema ventralis*, *Halyomorpha halys*, or *Nezara viridula*. Control consisted of clean air. *N* number of responding insects. *N.R.* number of not-responding insects (discarded from the analysis). Planned comparisons were tested within GLM with Gaussian error distribution (residence time) or with binomial error distribution (first choice).

Females of *T. mitsukurii* did not exhibit any attraction towards *H. halys* (*P* = 0.85), or any of the native stink bugs, as residence times in treatment and control did not differ (*P* ≥ 0.11 for all the comparisons) (Fig. 3, Table S5). First choice analysis confirmed the absence of a significant attractivity of the eggs of all stink bug species tested (*P* ≥ 0.13 for all comparisons) (Fig. 3, Table S6).

## Discussion

Among the different stink bug-associated odours tested in the olfactometer, only those from plants bearing an egg mass of *H. halys* or *N. viridula* elicited positive attraction in *T. mitsukurii* females. These results validated our hypothesis that a more in-depth evaluation of the parasitoid host specificity, through chemical ecology investigation, would further restrict the parasitoid host range that was depicted by physiological host range assays. Indeed, several non-coevolved hosts, although accepted under laboratory simplified conditions (i.e., no-choice and paired choice black-box tests), would be hardly located in the field due to a lack of suitable host-associated odours. This interpretation conceptually agrees with results from prior studies on *T. japonicus*, another *H. halys* exotic biocontrol agent that is currently being released in Italy^28^. In fact, *T. japonicus* displayed a relatively wide physiological host range in laboratory choice and no-choice assays^6,8,29^, but a more restricted oligophagy in olfactometer or in field conditions^44,53^.

Our results also indicate that soybean plants exposed to *H. halys* feeding and egg deposition can emit volatile molecules that are detected by the coevolved parasitoid *T. mitsukurii*. This response is consistent among residence time and first choice data. Volatile emission induced by host oviposition represents an indirect defence for the plants, as already demonstrated in different systems involving *Trissolcus* egg parasitoids^44,45,48^, and is an exceptionally reliable signal of the presence of target hosts in the canopy^9,11^. At the plant physiological level, induced defences have been demonstrated to involve JA- and/or SA-defensive pathways, depending on stink bug species, type and timing of attack^54–57^. Induced plant volatiles act on long-distance range and are easily detected by the egg parasitoids, allowing them to rapidly locate the host that is suitable only for a short period^45,58^. The ability of *T. mitsukurii* to exploit induced plant volatiles for locating *H. halys*, although it is investigated here for the first time, was recently hypothesized following results of open field surveys in northeast Italy and France^41,59^. For instance, higher parasitization of *H. halys* by *T. mitsukurii* was detected in naturally-laid eggs compared to sentinel eggs^59^. In two other studies, *T. mitsukurii* exhibited remarkable discovery efficiency of *H.halys* eggs laid on plant tissues, with observed parasitisation on egg masses that ranged from 27.3 to 46.5%^35,41^.

Noteworthy is also the positive attraction of *T. mitsukurii* towards plants bearing eggs of the non-coevolved *N. viridula.* Our result, together with the fact that *T. mitsukurii* positively responds to tracks of *N. viridula* females^60^, would suggest high ability to locate eggs. Indeed, the parasitoid is considered a main enemy of *N. viridula* in Japanese areas where the stink bug has been established^37^. Surprisingly, despite this remarked discovery ability, *T. mitsukurii* rarely emerged (successful development and emergence from host eggs) from laboratory-reared or field-collected eggs of *N. viridula* in the case of adventive Italian population(s)^35,43^. Conversely, emergence of *T. mitsukurii* strains from *N. viridula* eggs in paddy fields or early-planting rice in Japan was variable (e.g., 12 to 51% of emerged offspring calculated on the total number of eggs)^61,62^. The mismatch between the positive response of *T. mitsukurii* to cues associated with *N. viridula* and the inconstant suitability of this host for parasitoid development is partially consistent with lack of coevolution, as *N. viridula* is of Ethiopian-South Mediterranean origin^63^. However, this does not explain why the parasitoid responds to cues associated with the novel host. A hypothetical explanation for this could be that *N. viridula* induces in soybean a defensive response that might be similar to that induced by other herbivores, like *Nezara antennata* Scott, native to Eastern Asia^51^ and listed as host of *T. mitsukurii*^40^. It is known that *N. viridula* and *N. antennata* share common volatile compounds^47^, however, whether they also induce similar plant responses is unknown.

Previous choice and no-choice bioassays underlined high acceptance and suitability of *D. baccarum* for *T. mitsukurii*^43^. Surprisingly, our data did not reveal any behavioural response of this parasitoid to odours from soybean plants bearing egg masses of *D. baccarum*. *Dolycoris baccarum* has a wide distribution throughout the Palearctic region^51^, including Asia where it is a pest of several crops including soybean^64,65^. Although *D. baccarum* is listed in the host range of *T. mitsukurii*^40^, there are no quantitative data related to the prevalence rate of this parasitoid. On the other hand, naturally-laid and sentinel egg masses of *D. baccarum* are highly parasitized in Chinese orchards by other parasitoids, i.e., *T. japonicus*^29^. Similarly, in Korea and Japan, *Trissolcus nigripedius* Nakagawa and *Telenomus gifuensis* Ashmead (both Hymenoptera: Scelionidae) are commonly found parasitizing *D. baccarum* eggs laid in crop fields, including soybean^66^, sometimes demonstrating high prevalence^67^. Therefore, while some parasitoid species seem to effectively track host eggs of *D. baccarum* in the field, the same cannot be demonstrated for *T. mitsukurii*, suggesting that parasitization by this species might be occasional.

In no-choice black box experiments, *E. ventralis* eggs represented a poorly suitable host for *T. mitsukurii*, as parasitoids failed to develop inside its eggs^43^. The lack of response towards odours from plants bearing an egg mass of *E. ventralis* in our experiments is consistent with the very low suitability of this species^43^. The stink bug is of West Palearctic origin and is only marginally present in the native area of *T. mitsukurii*^52^. Therefore, the risk that under field conditions *T. mitsukurii* would parasitize *E. ventralis* appears quite low.

One of the most desired aspects of risk assessment is that the candidate biocontrol agent has no or limited negative effect on beneficials. The fact that plants with *A. custos* eggs were less preferred by *T. mitsukurii* females compared to the control can be interpreted as a form of repellence towards the treatment odour^19,68^ and this may reduce the risk of non-target parasitisation. We may expect that in case of intentional release of *T. mitsukurii*, the existence of such an ecological barrier would limit encounters with *A. custos* eggs in the field. The response of *T. mitsukurii* to *A. custos* appears at least partly similar to that of *T. japonicus*, which in no-choice tests successfully parasitized this stink bug predator, but in large cage tests, using plant bearing egg masses, preferred *H. halys* over *A. custos*^8,43^. Similarly to herbivorous species, zoophytophagous Heteroptera, including *A. custos*, can feed on plant tissues to acquire water and nutrients^69^. It was demonstrated that some of these species can induce the activation of defensive signalling pathways in plants, with consequent release of volatile organic compounds, which can inform natural enemies of the ongoing attack^70,71^. Eventually, behavioural responses of natural enemies to such odour sources can vary in different systems. For instance, oviposition by the zoophytophagous *Podisus maculiventris* (Say) (Hemiptera: Pentatomidae) induces the emission of plant volatiles which attract its coevolved parasitoid *Telenomus podisi* (Ashmead) (Hymenoptera: Scelionidae)^71^. In another trophic system, oviposition by the predatory *P. maculiventris* on tomato plants did not attract *T. japonicus*, although the parasitoid is able to successfully parasitize *P. maculiventris* eggs and develop inside the host^44,72^. The lack of a coevolutionary history between the two species may explain the inconsistent behaviour of *T. japonicus*^44^. *Arma custos* (junior synonym *Arma chinensis* Fallou^73^) is historically present in *T. mitsukurii*’s area of origin^74^, hence we can speculate that the observed avoidance can be interpreted as a coevolutionary adaptation within the tritrophic system that might prevent the parasitoid to exploit the predator. Although *A. custos* eggs were highly suitable for *T. mitsukurii* in no-choice black box tests^43^, development of the parasitoid larva is highly risky due to possible predatory (cannibalistic) behaviour of *A. custos* nymphs and adults, and considering that parasitized eggs tend to remain for a longer time compared to viable eggs^74–76^. Hence, in the first step of the hierarchical process of habitat assessment, the parasitoid could use plant odours to avoid such a risky host. *Halyomorpha halys* viable eggs could be also exposed to cannibalism, but the stink-bug appeared to have developed a strategy to synchronize egg hatching in order to prevent sibling cannibalism^77^.

The exploitation of adult-related chemical odours is quite common in egg parasitoids^11,78,79^, however in the present study we noticed a lack of *T. mitsukurii* response to females of *H. halys*. Although it is known that volatile and non-volatile cues from physogastric females may represent reliable information for some Scelionid wasps, kairomones from stink bug females elicit parasitoid responses mainly at short distance^47,80^. For instance, a previous investigation demonstrated that *T. japonicus* responds to *H. halys* females only in a “short-distance” olfactometer and not in a “long-distance” olfactometer (as the one we have used here)^44^. In open arenas, *Trissolcus brochymenae* (Ashmead) (Hymenoptera: Scelionidae) responded to *Murgantia histrionica* (Hahn) (Hemiptera: Pentatomidae) physogastric females but not to males^81^. Concerning *T. mitsukurii*, female extracts of *H. halys* seem to elicit a behavioural response of the parasitoid in Petri dish arenas, thus in a short-distance environment^60^.

We did not detect any attraction of *T. mitsukurii* towards odours from eggs of the tested stink bugs. Kairomones from host eggs are typically present in small amounts, hence their role in host location is mainly expected at short distance (reviewed by^11,82,83^). In olfactometers, even though few species of egg parasitoids (e.g., *T. podisi*) were demonstrated to respond to odours directly emitted by eggs^84^, so far, investigated *Trissolcus* species did not respond^44^ except when a high number of eggs was placed very close to the air hole at the end of the olfactometer arm^81^. Hence, we can hypothesize that such poorly detectable odours would only permit host location in close proximity, as shown for *T. brochymenae* using short-range bioassays in open arenas^81^.

In conclusion, while previous host-acceptance investigations revealed a broad host range for *T. mitsukurii* in Europe^43^, present results suggest that host location at long distance would likely favour parasitization of *H. halys* (or *N. viridula*), rather than the other stink bugs tested here, in particular the beneficial *A. custos*. Possibly, the presence of such an ecological filter would have positive implications for preventing undesired impacts on non-targets in case of intentional release of the parasitoid in biological control programs. On the other hand, there are no physiological impediments for *T. mitsukurii* to develop in some non-targets (e.g., *A. custos*), hence when such species co-occur in the field with *H. halys*, they would likely be more exposed to parasitization due to occasional encounters during foraging. Hence, the bioassays conducted here with *T. mitsukurii* following the host range studies^43^, highlight the need of a multidisciplinary approach in pre-release risk assessment, where results from each step are part of the puzzle that will allow a reliable field scenario prediction. Dedicated field surveys of native and exotic stink bug eggs in those areas where *T. mitsukurii* have fortuitously established would likely help in estimating the relevance of such host-parasitoid interactions and provide better support for the definition of a risk assessment document, necessary for licensing parasitoid releases.

## Methods

### Origin of insects and rearing

Stink bug colonies were established from adults collected in spring and summer 2020 in Northern and Central Italy from fruit orchards, herbaceous crops and uncultivated areas. Adult stink bugs were collected by sweep netting or visual handpicking on grasses, bushes and trees. Field collected adults were transferred to the laboratory and reared in insect cages (BugDorm 4F4545, Insect MegaView Science Co. Ltd., Taichung, Taiwan) under environmentally controlled conditions (25 ± 1 °C, 60 ± 5% RH and 16:8 h L:D). Phytophagous stink bugs were maintained with a mixed diet based on fruits, vegetables and seeds, whereas the predatory *A. custos* was fed with *Tenebrio molitor* L. pupae (Coleoptera: Tenebrionidae). Food was replaced three times per week. A daily wetted cotton wool placed inside an opened Petri dish (9 cm diam.) was used for water provision. About 10 paper towels (20 cm × 20 cm) were added inside each rearing cage to provide an oviposition substrate.

*Trissolcus mitsukurii* populations were initiated from *H. halys* parasitized egg masses originally collected in fruit orchards in north-eastern Italy. The parasitoid colony was replenished yearly with new field-collected specimens^34^. For breeding maintenance, 1-d-old *H. halys* egg masses were exposed to a parasitoid female for 24 h. The wasps were held in glass tubes (2 cm diam. × 15 cm length) sealed on both sides with a plastic mesh. A diet of honey droplets was dispensed on a rectangular cardboard (2 cm large × 4 cm length), offered to parasitoids and replenished two times per week. Males and females were kept together to permit mating and 7-day-old females were isolated in glass tubes before bioassays. Maintenance of the egg parasitoid colony (at 25 ± 1 °C, 60 ± 5% and 16:8 h L:D) and all olfactometer bioassays (see below) were conducted under officially authorized quarantine conditions at CREA facilities (DG/DISR/DISR05/0013647-19/04/2018).

### Plant rearing and exposure for stink bug oviposition

Seeds of soybean, *Glycine max* (L.) Merrill, and cauliflower, *Brassica oleracea* var. *botrytis* L., were sown in plastic pots (6.5 cm height × 5.5 cm diam. at mid-height) containing a horticultural substrate (commercial name Radicom, Vigorplant Italia S.r.l., Fombio, Italy). Plants were grown and maintained in a rearing room (24 ± 2 °C, 55 ± 10% RH, 16: 8 h L:D) and irrigated every 2 d. A mixture (1.4 g/L) of fertilizer (5-15-45, NPK, Plantfol, Valagro, Italia) was added one week after plant emergence. Two plants, 2- to 4-weeks old (approx. 15–20 cm tall) depending on plant species, were placed inside an insect cage containing mated females of each colony of the stink bug species tested. Soybean plants were used for *A. custos*, *D. baccarum*, *H. halys*, and *N. viridula,* while cauliflower plants were used for *E. ventralis*, as this species has preference for crucifers^85^. Plants were checked twice per day for the presence of egg masses laid on the abaxial leaf surface. Whenever eggs were detected, plants were removed from the cages and tested for their attractiveness within 24 h. If no eggs were found after 3 d of exposure, plants were replaced with new clean plants.

### Odour sources tested in the bioassays

Using a Y-tube olfactometer we tested the behavioural responses of *T. mitsukurii* to plant-host odours associated with native and non-native stink bugs. For each of the five stink bug species, i.e., *A. custos*, *D. baccarum*, *E. ventralis*, *H. halys,* and *N. viridula*, the following treatments were evaluated:

- *Plant* + *Eggs*: a plant exposed to stink bug feeding and bearing 1 naturally laid egg mass (< 24 h old);
- *Females*: a batch of four females in the ovipositional phase, i.e., with physogastric abdomen ^79^;
- *Eggs*: about 100 eggs < 24 h old. Only egg masses laid on unbleached paper towels were tested.

Controls consisted of clean air (AIR) for treatments with females and eggs, or of an insect-unexposed soybean or cauliflower plant (CLEAN PLANT) for treatments with plants.

### Olfactometer bioassays

The female behavioural responses to the different odour stimuli were investigated in a Y-tube olfactometer (common stem: 90 mm length; arms: 80 mm length each at 100° angle between arms and equally distant from the common stem, internal section: 15 mm × 10 mm), carved in a plexiglass plate (200 mm × 190 mm × 10 mm thick) and sandwiched between two glass sheaths (each plate: 200 mm × 150 mm × 5 mm thick), which provided the upper and lower closure of the olfactometer^79^. Two identical Y-tube olfactometers were simultaneously used, allowing to observe the behaviour of two parasitoids at the same time. A stream of environmental air was inflated by a diaphragm pump (KNF Italia S.r.l., Milan, Italy) through an activated charcoal filter (260 mm length × 40 mm internal diam.). The airflow was humidified through a Dreschel bottle (250 mL volume) containing distilled water and split into two identical routes, each consisting of a flowmeter that regulated the airflow at 0.8 L/min and a glass chamber (30 cm height × 10 cm internal diam., ~ 1.9 L volume) containing the odour source (treatment or control cue). The glass chamber was sealed at the base to a Teflon disk (7 mm height, 14 cm diam.) using Parafilm M® sealing film (Heathrow Scientific, Vernon Hills, IL, USA). The airflow was conveyed using tubes in Silicone (6 mm internal diam.). Tubes were connected to the chamber by means of plastic opened screw caps (Kartell Spa, Noviglo, Italy). The airflow coming out from the glass chamber was eventually split again into two and conveyed to one of the two arms of both olfactometers. A digital flowmeter (mod. GFM17, Aalborg, New York, USA) was used to measure the flow rate entering each olfactometer arm (~ 200 mL/min). The olfactometer device was surrounded by a black fabric curtain to minimize external cues from the room and was illuminated by two 36 W cool white fluorescent tubes located above the device.

About 30 min before the bioassays, parasitoids, stink bugs and plants were moved to the bioassay room, maintained at 25 °C, to acclimatize. Bioassays were conducted from 09:00 to 16:00. In detail, a parasitoid female was introduced into the central stem of the Y-tube and the behaviour was recorded. After 4 bioassays the position of the tubes entering the Y-tube arms was switched to avoid possible bias. Additionally, the glass plates were cleaned with a laboratory detergent (2% solution of Cleanilab LM1; Kartell Spa, Noviglo, Italy), rinsed with tap water and acetone, whereas the plexiglass part of the olfactometer was cleaned with detergent, rinsed with tap water, and finally rinsed with distilled water. Each *T. mitsukurii* female was observed for 10 min. Parasitoid females were evaluated once. The time spent in each olfactometer arm and in the common stem was visually recorded with JWatcher 1.0^86,87^. For each treatment, depending on insect and egg mass availability, 3–4 replicates of the same odour stimulus (plant bearing an egg mass, stink bug females, or eggs) were tested. For the different stink-bug species, 56 to 77 parasitoids were eventually evaluated for each treatment with a plant bearing an egg mass, 44 to 70 parasitoids for stink bug female tests, and 32 to 69 insects for the stink bug eggs tests.

### Statistical analyses

The walking behaviour of the parasitoid in the olfactometer was described by the residence time, i.e., the time spent by the female in each olfactometer arm, and by the first choice, i.e., the olfactometer arm the parasitoid entered first. Females that did not make a choice or only entered either olfactometer arm for a limited time (< 30 s) were considered not responding and were discarded from the analysis (similar to^88^). For the analysis, the logarithmic transformation of the ratio between the residence time in the treatment arm versus the residence time in the control arm was calculated. This transformation (log-ratio) ensured that only one measure per insect was later analysed^89^. Generalized linear models (GLMs) with Gaussian error distribution (for residence time data) or with binomial error distribution (for first choice data) were fitted to test differences of treatment versus control within each odour source. Analyses were conducted in the R statistical environment, version 4.0.2^90^.

## Supplementary Information

Supplementary Tables.

## Data Availability

The data generated during the current study are available from the corresponding author on request.
