# Supplementary material for: Olfactory responses of Trissolcus mitsukurii to plants attacked by target and non-target stink bugs suggest low risk for biological control
Source: Sci Rep. 2022 Feb 3;12:1880. doi: 10.1038/s41598-022-05873-w (PMC8814161; doi:10.1038/s41598-022-05873-w)
Supplement: Supplementary file 1 — Supplementary Tables. [file 41598_2022_5873_MOESM1_ESM.docx]

**OLFACTORY RESPONSES OF *TRISSOLCUS MITSUKURII* TO PLANTS ATTACKED BY TARGET AND NON-TARGET STINK BUGS SUGGEST LOW RISK FOR BIOLOGICAL CONTROL**

Gabriele Rondoni^1*^, Elena Chierici^1^, Lucrezia Giovannini^2^, Giuseppino Sabbatini-Peverieri^2^, Pio Federico Roversi^2^, Eric Conti^1^

^1^ Department of Agricultural, Food and Environmental Sciences, University of Perugia, Perugia, Italy

^2^ CREA – Research Centre for Plant Protection and Certification, Florence, Italy

*Corresponding author: [gg.rondoni@gmail.com](mailto:gg.rondoni@gmail.com)

Supplementary Table S1: Summary of the hypotheses tested within Gaussian GLM on the residence time of *Trissolcus mitsukurii* exposed to odours from plant with an eggs mass of *Arma custos* (AC), *Dolycoris baccarum* (DB), *Eurydema ventralis* (EV), *Halyomorpha halys* (HH), or *Nezara viridula* (NV). Control consisted of a clean soybean or cauliflower plant (CP). For each comparison, the estimated coefficient (the log ratio between the treatment and the control residence time), SE, t-value and P-value are reported.

| Planned comparisons | Estimate | SE | t value | P |  |
| --- | --- | --- | --- | --- | --- |
| AC vs CP | -1.71 | 0.77 | -2.21 | 0.0275 | * |
| DB vs CP | -0.79 | 0.70 | -1.13 | 0.2596 | ns |
| EV vs CP | 0.12 | 0.77 | 0.15 | 0.8793 | ns |
| HH vs CP | 1.82 | 0.71 | 2.54 | 0.0115 | * |
| NV vs CP | 1.80 | 0.82 | 2.20 | 0.0288 | * |
| Significance: ‘*’ P ≤ 0.05, ‘ns’ P > 0.05 | | | | | |

Supplementary Table S2: Summary of the hypotheses tested within binomial GLM on the first choice of *Trissolcus mitsukurii* exposed to odours from plant with an eggs mass of *Arma custos* (AC), *Dolycoris baccarum* (DB), *Eurydema ventralis* (EV), *Halyomorpha halys* (HH), or *Nezara viridula* (NV). Control consisted of a clean soybean or cauliflower plant (CP). For each comparison, the estimate coefficient (log odd), SE, z-value and P-value are reported.

| Planned comparisons | Estimate | SE | z value | P |  |  |
| --- | --- | --- | --- | --- | --- | --- |
| AC vs CP | -0.35 | 0.26 | -1.38 | 0.16798 | ns |  |
| DB vs CP | 0.078 | 0.23 | 0.34 | 0.73251 | ns |  |
| EV vs CP | -0.063 | 0.25 | -0.25 | 0.80262 | ns |  |
| HH vs CP | 0.67 | 0.25 | 2.74 | 0.00618 | ** |  |
| NV vs CP | 0.22 | 0.27 | 0.80 | 0.42357 | ns |  |
| Significance: ‘**’ P ≤ 0.00, ‘ns’ P > 0.05 | | | | | | |

Supplementary Table S3: Summary of the hypotheses tested within Gaussian GLM on the residence time of *Trissolcus mitsukurii* exposed to odours from stink bug females of *Arma custos* (AC), *Dolycoris baccarum* (DB), *Eurydema ventralis* (EV), *Halyomorpha halys* (HH), or *Nezara viridula* (NV). Control consisted of clean air (AIR). For each comparison, the estimated coefficient (the log ratio between the treatment and the control residence time), SE, t-value and P-value are reported.

| Planned comparisons | Estimate | SE | t value | P |  |
| --- | --- | --- | --- | --- | --- |
| AC vs AIR | -1.031 | 1.087 | -0.95 | 0.344 | ns |
| DB vs AIR | -0.68 | 1.02 | -0.67 | 0.505 | ns |
| EV vs AIR | -0.12 | 0.86 | -0.14 | 0.892 | ns |
| HH vs AIR | 0.058 | 0.92 | 0.063 | 0.950 | ns |
| NV vs AIR | -0.16 | 1.052 | -0.15 | 0.877 | ns |
| Significance: ‘ns’ P > 0.05 | | | | |  |

Supplementary Table S4: Summary of the hypotheses tested within the binomial GLM on the first choice of *Trissolcus mitsukurii* exposed to odours from stink bug females of *Arma custos* (AC), *Dolycoris baccarum* (DB), *Eurydema ventralis* (EV), *Halyomorpha halys* (HH), or *Nezara viridula* (NV). Control consisted of clean air (AIR). For each comparison, the estimate coefficient (log odd), SE, z-value and P-value are reported.

| Planned comparisons | Estimate | SE | z value | P |  |
| --- | --- | --- | --- | --- | --- |
| AC vs AIR | 0.00 | 0.30 | 0.00 | 1.000 | ns |
| DB vs AIR | -0.24 | 0.29 | -0.85 | 0.397 | ns |
| EV vs AIR | -0.17 | 0.24 | -0.72 | 0.474 | ns |
| HH vs AIR | 0.033 | 0.26 | 0.13 | 0.898 | ns |
| NV vs AIR | -0.13 | 0.29 | -0.44 | 0.662 | ns |
| Significance: ‘ns’ P > 0.05 | | | | |  |

Supplementary Table S5: Summary of the hypotheses tested within Gaussian GLM on the residence time of *Trissolcus mitsukurii* exposed to odours from stink bug eggs of *Arma custos* (AC), *Dolycoris baccarum* (DB), *Eurydema ventralis* (EV), *Halyomorpha halys* (HH), or *Nezara viridula* (NV). Control consisted of clean air (AIR). For each comparison, the estimated coefficient (the log ratio between the treatment and the control residence time), SE, t-value and P-value are reported.

| Planned comparisons | Estimate | SE | t value | P |  |
| --- | --- | --- | --- | --- | --- |
| AC vs AIR | 0.66 | 0.99 | 0.66 | 0.509 | ns |
| DB vs AIR | -1.3 | 0.81 | -1.61 | 0.110 | ns |
| EV vs AIR | 1.10 | 1.14 | 0.97 | 0.332 | ns |
| HH vs AIR | -0.17 | 0.92 | -0.18 | 0.855 | ns |
| NV vs AIR | -0.035 | 1.19 | -0.030 | 0.976 | ns |
| Significance: ‘ns’ P > 0.05 | | | | |  |

Supplementary Table S6: Summary of the hypotheses tested within the binomial GLM on the first choice of *Trissolcus mitsukurii* exposed to odours from stink bug eggs of *Arma custos* (AC), *Dolycoris baccarum* (DB), *Eurydema ventralis* (EV), *Halyomorpha halys* (HH), or *Nezara viridula* (NV). Control consisted of clean air (AIR). For each comparison, the estimate coefficient (log odd), SE, z-value and P-value are reported.

| Planned comparisons | Estimate | SE | z value | P |  |
| --- | --- | --- | --- | --- | --- |
| AC vs AIR | 0.00 | 0.30 | 0.00 | 1.000 | ns |
| DB vs AIR | -0.26 | 0.24 | -1.080 | 0.280 | ns |
| EV vs AIR | 0.53 | 0.35 | 1.50 | 0.133 | ns |
| HH vs AIR | 0.0074 | 0.27 | 0.27 | 0.786 | ns |
| NV vs AIR | 0.00 | 0.35 | 0.00 | 1.000 | ns |
| Significance: ‘ns’ P > 0.05 | | | | | |
